# Metabolomic Investigation of Ultraviolet Ray-Inactivated White Spot Syndrome Virus-Induced Trained Immunity in *Marsupenaeus japonicus*

**DOI:** 10.3389/fimmu.2022.885782

**Published:** 2022-05-26

**Authors:** Shaoqing Zang, Li-Xia Lv, Chen-Fei Liu, Peng Zhang, Cang Li, Jin-Xing Wang

**Affiliations:** ^1^Shandong Provincial Key Laboratory of Animal Cells and Developmental Biology, School of Life Sciences, Shandong University, Qingdao, China; ^2^State Key Laboratory of Microbial Technology, Shandong University, Qingdao, China

**Keywords:** trained immunity, shrimp, WSSV, GC–MS/MS, metabolites

## Abstract

Trained immunity is driven by metabolism and epigenetics in innate immune cells in mammals. The phenomenon of trained immunity has been identified in invertebrates, including shrimp, but the underlying mechanisms remain unclear. To elucidate mechanisms of trained immunity in shrimp, the metabolomic changes in hemolymph of *Marsupenaeus japonicus* trained by the UV-inactivated white spot syndrome virus (UV-WSSV) were analyzed using tandem gas chromatography–mass/mass spectrometry. The metabolomic profiles of shrimp trained with UV-WSSV followed WSSV infection showed significant differences comparison with the control groups, PBS injection followed WSSV infection. 16 differential metabolites in total of 154 metabolites were identified, including D-fructose-6-phosphate, D-glucose-6-phosphate, and D-fructose-6-phosphate, and metabolic pathways, glycolysis, pentose phosphate pathway, and AMPK signaling pathway were enriched in the UV-WSSV trained groups. Further study found that histone monomethylation and trimethylation at H3K4 (H3K4me1 and H3K4me3) were involved in the trained immunity. Our data suggest that the UV-WSSV induced trained immunity leads to metabolism reprogramming in the shrimp and provide insights for WSSV control in shrimp aquaculture.

## Introduction

The host immune response can be classified into innate and adaptive immune responses according to the classical immune theory. Vertebrates have both innate and adaptive immunity, while invertebrates have only the former (1). It is generally believed that adaptive immunity generates specific immunological memory, and innate immunity has no memory. However, a large body of research evidence has challenged this dogma. In organisms lacking adaptive immunity as well as in mammals, the innate immune system can mount resistance to reinfection, this phenomenon termed trained immunity or innate immune memory (2–4). Briefly, the first stimulus to host innate immune cells by pathogen-associated molecular patterns leads to changes in the functional immune status; subsequently, the immune activation status returns to the basal level during the resting phase (3). At the second homologous or heterologous encounter, the functions of cells against pathogens are enhanced at much a faster and higher level than those induced during the first challenge (2, 4). Trained immunity has been reported in invertebrates, such as shrimp and crayfish (5–8), and in plants (9).

While specific pathways and markers differ between the various adaptive programs (including differentiation, priming, trained immunity and tolerance according to functional state of innate immune cells) in innate immunity, they all use the same basic mechanisms (epigenetic, transcriptional and metabolic), but with different flavors (10). The core mechanism underlying trained immunity is the interaction between epigenetic modification and metabolism. In mammals, changes in the metabolism of trained innate immune cells are closely related to epigenetics (11). When stimulated by training components, the metabolome of innate immune cells is reprogrammed; further, changes in metabolites activate epigenetic modifications, thereby inducing changes in chromatin. Chromatin is specifically modified and the expression of immune-related genes is then activated (3). In trained immunity, although the cell immune response reverts to a low level at the resting phase and several epigenetic modifications of chromatin are lost, the regulatory region retains the activated modification partially as a potential enhancer for a long time to exist. Thus, by rapidly modifying chromatin at the same site to activate immune-related genes, cells can strongly and quickly respond to secondary infections (3, 12). Several studies exist on the mechanisms underlying trained immunity in mammals; nevertheless, the molecular mechanisms linking immunological signals induced by microbial stimuli to epigenetic changes have not been clearly revealed.

Metabolomics is widely used to study the metabolic network of a biological system by investigating metabolite profiles before and after the system is stimulated or disturbed. In recent years, it has been extensively used for studying aquaculture diseases so as to develop novel strategies for their prevention and control (13). In the context of trained immunity, increasing evidence has associated metabolism to epigenetic modifications (14). Several metabolites involved in glycolysis and the tricarboxylic acid (TCA) cycle can serve as cofactors for epigenetic writers and erasers, such as DNA and histone methyltransferases and demethylases and histone acetyltransferases and deacetylases (11, 15). Although the phenomenon of trained immunity has been reported in shrimp, pertinent molecular mechanisms remain almost completely unclear. Based on growing evidences of metabolites with epigenetic modification, we applied metabolic analysis to study the mechanism of shrimp trained immunity.

Shrimp is an economically important animal in marine aquaculture. It is highly vulnerable to diseases, particularly the white spot syndrome caused by white spot syndrome virus (WSSV); such problems lead to heavy losses to the shrimp farming industry. Despite this, effective prevention and control measures remain missing. To tackle this problem, we established a trained immunity model induced by UV-inactivated white spot syndrome virus (UV-WSSV) in kuruma shrimp *Marsupenaeus japonicus* and performed a metabolomic-based analysis to elucidate the mechanisms underlying trained immunity in shrimp. By comparing changes in the metabolome, we identified key metabolites and metabolic pathways associated with trained immunity in shrimp, which might be related to epigenetic modifications. The results reported herein can not only serve as a theoretical basis for trained immunity in shrimp but also facilitate disease prevention and control in aquaculture.

## Results

### UV-WSSV Induced Trained Immunity in *M. japonicus*

**Figure 1:**
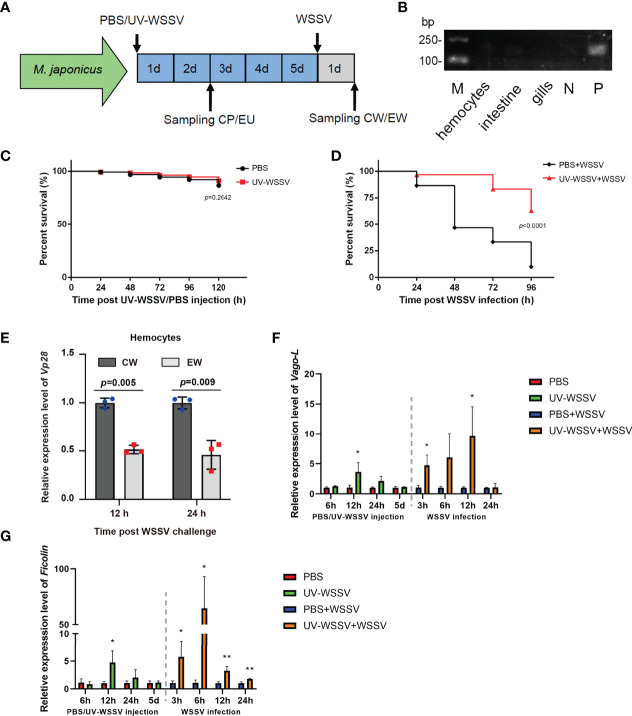
Trained immunity model. **(A)** UV-WSSV-induced trained immunity in *M. japonicus*. UV-WSSV (50 μL, 5 × 10^5^ copies) was injected into shrimp; the same volume of PBS was injected into controls. Five days after the first injection (training), a second injection of WSSV was administered (50 μL, 5 × 10^5^ copies). Hemolymph was collected at 48 h after the first injection (CP, PBS-injected shrimp; EU, UV-WSSV-injected shrimp) and 24 h after the second one (CW, PBS-injected shrimp infected with WSSV; EW, UV-WSSV-injected shrimp infected with WSSV). **(B)** *Vp28* expression in hemocytes, intestine and gills of shrimp analyzed by RT-PCR. N was negative control (ddH2O). P was positive control (WSSV). **(C)** Survival rate analysis of shrimp injected with UV-WSSV; controls were injected with an equal volume of sterile PBS. **(D)** Survival rate of shrimp, which was analyzed using the log-rank (Mantel–Cox) test. Normal shrimp were divided into two groups, and the experimental group was first trained with UV-WSSV, and then live WSSV was injected after 5 days. In case of the control group, an equal volume of sterile PBS was first injected, and then the same amount of WSSV was administered. **(E)** WSSV replication was analyzed using *Vp28* as an indicator in hemocytes of control and UV-WSSV trained shrimp at 12 and 24 h post WSSV infection (the second infection). The shrimp injected with a corresponding amount of PBS in training period and then infected with WSSV were used as controls. **(F, G)** The expression patterns of *Vago-L* **(F)** and *Ficolin* **(G)** in hemocytes of shrimp in the UV-WSSV trained immunity model. Student’s *t* test was used for statistical analysis, and *p* < 0.05 was considered to demonstrate statistically significant differences. *p < 0.05, **p < 0.01.

We first established a training model using UV-WSSV (**Figure 1A**) in *M. japonicus*. Before training with UV-WSSV/PBS, we firstly detected if the shrimp were free from WSSV infection, using *Vp28* as an indicator by reverse transcription-PCR (RT-PCR). As show in **Figure 1B**, *Vp28* expression was not detected in hemocytes, intestine and gills of shrimp randomly selected and double distilled water (ddH_2_O) (a negative control), but was detected in the positive control (WSSV). The result suggested that the untreated shrimp were free from WSSV infection (**Figure 1B**).

To test whether UV-WSSV was completely inactivated upon UV treatment, the survival rate of shrimp was analyzed. We observed that the survival rate of shrimp injected with UV-WSSV (5 × 10^5^) was the same as that of those injected with PBS (**Figure 1C**), suggesting that WSSV was completely inactivated. Further, 5 days after UV-WSSV injection, we infected shrimp with live WSSV. On assessing their survival rate, we found that the survival rate of UV-WSSV trained immunity shrimp was significantly higher than that of control (PBS injected) shrimp (**Figure 1D**), indicating that UV-WSSV enhanced the ability of shrimp to resist viral infections by inducing trained immunity.

To confirm above results, we analyzed WSSV replication using *Vp28* expression as an indicator in hemocytes of *M. japonicus* with quantitative real-time PCR (qRT-PCR) in the trained immunity model, and the result showed that *Vp28* expression was significantly lower in the UV-WSSV trained group than that in the PBS control group at 12 and 24 h post WSSV infection (**Figure 1E**). We also detected the expression patterns of some immune effector genes such as *Vago-like* (*Vago-L*) and *Ficolin*, and the results showed that during the training period with UV-WSSV, the expression of *Vago-L* and *Ficolin* were significantly upregulated in the shrimp at 12 h post UV-WSSV training compared with the PBS control, at the 5th day of UV-WSSV training, the expression of above two genes returned to a low level (at resting time), and then upregulated at 3 h post WSSV injection (the second challenge) in the UV-WSSV trained group (**Figures 1F, G**), which was consistent with the important characteristics of trained immunity, that is, faster and stronger expression in the second period of trained immunity (10). All the results suggested that UV-WSSV was able to induced trained immunity in *M. japonicus*.

### Multivariate Analysis of Metabolite Profiles

Metabolomic analysis was performed to explore the alterations in metabolism in UV-WSSV trained and control shrimp. GC–MS/MS was used for the metabolic profiling of plasma samples collected from various groups (CP, control group injected with PBS; EU, experimental group injected with UV-WSSV; CW, the control injected with WSSV and EW, UV-WSSV trained immunity group injected with WSSV) (**Supplementary Figure 1**). Subsequently, data were compared to identify differential metabolites (DMs) and associated metabolic pathways. Based on the classification of metabolites in Kyoto Encyclopedia of Genes and Genomes (KEGG) and Metabolon.inc, metabolites were classified by function; the largest category was “carbohydrates” (20.779%), followed by “amino acids” (16.883%) and “nucleotide” (12.987%) (**Supplementary Figure 2**).

To detect variations in metabolites and metabolic pathways, we analyzed and compared DMs between the EU and CP, EW and CW, and EU and EW groups. Simultaneously, we assessed reliability of the CP and CW groups.

**Figure 2:**
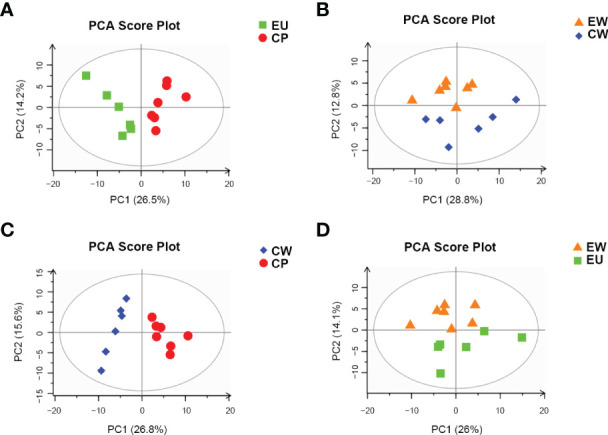
PCA score plots based on pairwise comparisons between different groups. **(A)** EU vs. CP, **(B)** EW vs. CW, **(C)** CW vs. CP, and **(D)** EW vs. EU groups.

Multivariate data analysis was performed to detect metabolic differences and the discrete changes of metabolic data between various pairs of groups. principal component analysis (PCA) showed the degree of aggregation and dispersion between samples. As evident from **Figure 2**, PCA score plots for all pairs of groups were properly aggregated within a group and separated between the groups of the original state of the metabolome data. R2X represents the interpretability of the PCA model. In general, R2X > 0.5 indicates good model reliability. **Table 1** shows R2X values for different pairs of groups.

**Table 1:** PCA validation parameters of various pairwise comparison groups.

Group	R2X	Pre
EU vs. CP	0.516	3
EW vs. CW	0.526	3
EW vs. EU	0.522	3
CW vs. CP	0.533	3

R2X, interpretability of the model (for X variables); Pre, number of principal components.

**Figure 3:**
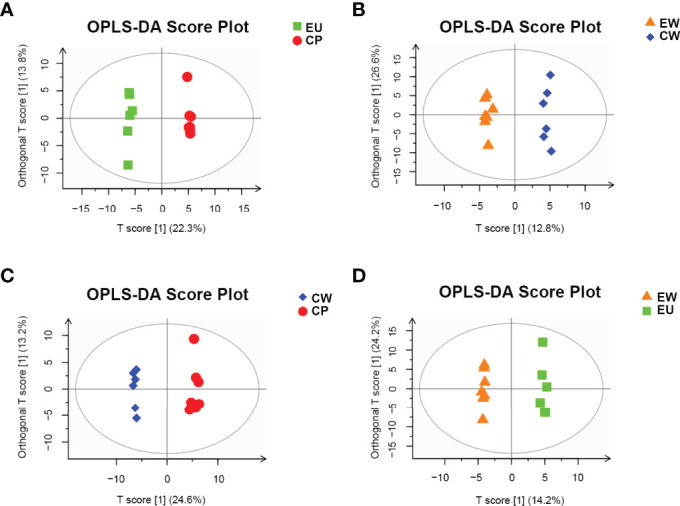
OPLS-DA score plots for pairwise comparisons between UV-WSSV trained immunity and control groups. **(A)** EU vs. CP, **(B)** EW vs. CW, **(C)** CW vs. CP, and **(D)** EW vs. EU groups.

We also constructed orthogonal projections to latent structures discriminant analysis (OPLS-DA) models to investigate the correlation between metabolite levels and conditions of different pairs of groups, with the OPLS-DA model representing the distribution of all datasets. As shown in **Figure 3**, obvious clusters were obtained upon comparing different pairs of groups. The PCA and OPLS-DA models nicely described the data of samples obtained from trained immunity in shrimp and could be used for further analyses of data.

**Figure 4:**
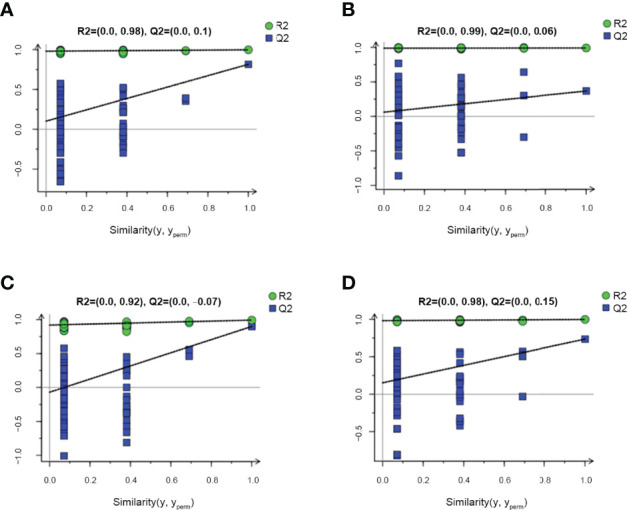
Permutation plots for pairwise comparisons between UV-WSSV trained immunity and control groups. **(A)** EU vs. CP, **(B)** EW vs. CW, **(C)** CW vs. CP, and **(D)** EW vs. EU groups.

A permutation plot can be used to effectively assess model overfitting. Herein the permutation plots for EU vs. CP, CW vs. CP, and EW vs. EU groups showed no overfitting, indicating that these models properly represented the samples and could be used for further data analyses (**Figures 4A, C, D**). In contrast, the permutation plot for EW vs. CW groups showed overfitting, suggesting that the data were invalid, and there were no significant differences in small molecules detected by GC–MS/MS (**Figure 4B**). Thus, no further analysis was performed for EW vs. CW group.

### Identification of DMs

Metabolomic analysis led to the identification of 154 metabolites. We compared changes in these metabolites based on variable importance in projection (VIP) score ≥ 1 obtained by OPLS-DA and *p* ≤ 0.05 (16, 17). Consequently, 45 and 23 DMs were identified in EU vs. CP and EW vs. EU groups, respectively. **Tables 2**, **3** show the classification, associated pathways, and fold-changes in DMs for EU vs. CP and EW vs. EU groups, respectively. DMs identified in CW vs. CP and EW vs. CW groups are listed in **Supplementary Tables 1****,** **2**, respectively.

**Table 2:** Significantly changed differential metabolites in EU vs. CP groups.

Categories	Compound	Pathway involved	Fold change	*p*-value	VIP
Amino acid	L-Isoleucine	Valine, leucine and isoleucine biosynthesis	4.714	0.00619	1.511
	L-Homoserine	Glycine, serine and threonine metabolism	4.427	0.0104	1.443
	Hypotaurine	Taurine and hypotaurine metabolism	4.141	0.00013	1.834
	N-Acetyl-beta-D-glucosamine	Amino sugar and nucleotide sugar metabolism	2.332	0.000919	1.704
	L-Threonine	Valine, leucine and isoleucine biosynthesis	1.873	0.0418	1.208
	Propylamine	Biosynthetic pathway of polyamines	1.684	0.00146	1.664
	Methyltetrahydrophenanthrenone	Amino acid metabolism	1.39	0.0447	1.195
	L-Pyroglutamic acid	Glutathione metabolism	1.252	0.000126	1.836
	Cadaverine	Protein digestion and absorption	0.609	9.60E-05	1.85
	L-Alanyl-L-alanine	Synthesis of the L-alanyl-L-alanine cross-bridge of peptidoglycan	0.226	0.0111	1.433
	L-Citrulline	Arginine biosynthesis	0.217	0.00951	1.455
	L-Valine	Valine, leucine and isoleucine biosynthesis	0.176	0.000359	1.773
	Urea	Arginine and proline metabolism	0.082	1.09E-06	2.003
	L-Ornithine	Arginine biosynthesis	0.069	0.00023	1.801
	L-Serine	Protein digestion and absorption	0.059	0.00348	1.578
	L-Leucine	Valine, leucine and isoleucine biosynthesis	0.041	0.0209	1.335
	L-Proline	Protein digestion and absorption	0.031	3.05E-06	1.978
	Taurine	Taurine and hypotaurine metabolism	0.02	0.00184	1.643
	L-Aspartic acid	Protein digestion and absorption	0.019	0.00127	1.676
Carbohydrate	D-Sorbitol	Galactose metabolism	1.551	0.0135	1.405
	D-Glucose-1-phosphate	Glucagon signaling pathway	1.511	0.00311	1.59
	2-O-(alpha-D-Mannosyl)-D-glycerate	Fructose and mannose metabolism	1.443	0.00521	1.532
	Methyl beta-D-galactoside	ABC transporters	1.318	0.0279	1.285
	Glycolate	Carbon metabolism	1.259	7.36E-06	1.953
	N-Acetyl-D-mannosamine	Amino sugar and nucleotide sugar metabolism	1.159	0.00492	1.539
	alpha-D-Glucose	Glycolysis/Gluconeogenesis	0.583	0.0288	1.279
Energy	Phosphoenolpyruvic acid	Tricarboxylic acid cycle, TCA cycle	1.63	0.021	1.335
	Malic acid	Central carbon metabolism in cancer	1.266	0.0155	1.384
	(1R,2S)-1-Hydroxypropane-1,2,3-tricarboxylate	Tricarboxylic acid cycle, TCA cycle	0.783	0.0403	1.216
Lipid	sn-Glycerol-1-phosphate	Glycerophospholipid metabolism	1.862	0.00543	1.527
	(9Z)-Octadecenoic acid	Biosynthesis of unsaturated fatty acids	1.701	0.0401	1.217
	Docosenoic acid	Biosynthesis of unsaturated fatty acids	1.7	0.0194	1.348
	Methanolphosphate	Biosynthesis of terpenoids and steroids	1.648	0.0254	1.302
	Arachidonate	Linoleic acid metabolism	1.623	0.0292	1.277
	Heptadecanoic acid	Synthesis of ferritin	1.292	0.00952	1.455
	1-Hexadecylglycerol	Absorption of chimyl alcohol	1.282	0.0309	1.267
	Dihydroxymalonic acid	Selective catalytic oxidation of glyceric acid	1.234	0.0141	1.398
	Linoleate	Linoleic acid metabolism	1.218	0.00564	1.523
	Hexadecanoic acid	Biosynthesis of unsaturated fatty acids	1.064	0.00581	1.519
	Squalene	Biosynthesis of alkaloids derived from terpenoid and polyketide	0.262	8.54E-06	1.949
Nucleotide	Uracil	beta-Alanine metabolism	0.125	0.00106	1.692
	Hypoxanthine	Purine metabolism	0.09	0.000198	1.811
Vitamin	4-Hydroxypyridine	4-hydroxypyridine catabolism	0.942	0.0316	1.263
Xenobiotics	Biphenyl	Degradation of biphenyl and polychlorinated biphenyl	0.918	0.0275	1.288
	Naphthalene	Naphthalene family	0.598	0.0167	1.372

**Table 3:** Significantly changed differential metabolites in EW vs. EU groups.

Categories	Compound	Pathway involved	Fold Change	*p*-value	VIP
Carbonic acid derivative	Urea	Arginine and proline metabolism	2.045	0.00139	2.094
Amino acid	Maleimide	Anabolism of alkaloids	1.427	0.0444	1.501
	Glycylglycine	Metabolic absorption of protein	1.286	0.00065	2.172
	Cadaverine	Lysine degradation	1.241	0.0134	1.763
	Propylamine	biosynthetic pathway of polyamines	1.139	0.0434	1.507
	L-Histidine	Biosynthesis of amino acids	0.756	0.0295	1.6
	L-Tyrosine	Biosynthesis of amino acids	0.653	0.0281	1.611
	L-Alanine	Biosynthesis of amino acids	0.578	0.0128	1.773
Carbohydrate	D-Glucose-1-phosphate	Glycolysis/Gluconeogenesis	0.759	0.0468	1.487
	beta-D-Glucose	Glycolysis/Gluconeogenesis	0.66	0.0467	1.488
	beta-D-Glucose-6-phosphate	Glycolysis/Gluconeogenesis	0.552	0.00359	1.976
	beta-D-Fructose-6-phosphate	Glycolysis/Gluconeogenesis	0.521	0.00445	1.946
	D-Fructose-6-Phosphate	Glucagon signaling pathway	0.517	9.86E-05	2.32
	L-Iditol	L-Iditol production from L-sorbose	0.434	0.00684	1.88
Carboxylic acid	Quinic acid	Phenylalanine, tyrosine and tryptophan biosynthesis	5.832	0.044	1.503
Energy	Succinate	Citrate cycle (TCA cycle)	1.427	0.000243	2.256
	Fumaric acid	Citrate cycle (TCA cycle)	1.332	0.00123	2.107
	(1R,2S)-1-Hydroxypropane-1,2,3-tricarboxylate	Tricarboxylic acid cycle	1.32	0.00968	1.823
	D-Ribose	Pentose phosphate pathway	0.667	0.0232	1.653
Lipid	1-Octanol	Microbial production of 1-octanol	1.132	0.0283	1.61
	Cholesterol	Cholesterol metabolism	0.555	0.0475	1.484
Nucleotide	Uridine	Pyrimidine metabolism	0.517	0.00198	2.052
Vitamin	3-Hydroxypyridine	Photooxidative stress	1.079	0.0223	1.662

#### Identification of DMs in EU vs. CP Groups

In EU vs. CP groups, 45 DMs were identified: 26 were upregulated and 19 were downregulated (**Table 2**). The top three most upregulated metabolites were L-isoleucine (4.71-fold), L-homoserine (4.42-fold), and hypotaurine (4.14-fold), whereas the top three most downregulated metabolites were L-proline, taurine, and L-aspartic acid. Based on *p* values and VIP scores, the most significantly altered metabolites were urea, L-proline, and glycolate; of them, glycolate was upregulated. Besides, as many as 14 types of amino acids were identified, including L-proline, L-pyroglutamic acid, L-ornithine, L-valine, and L-aspartic acid, accounting for a high proportion (>25%) of all DMs. Most of them were downregulated, accounting for 47% of all downregulated DMs. As evident from **Supplementary Figures 3A, B**, urea and L-proline were significantly downregulated in the EU group.

L-isoleucine is involved in protein metabolism, fatty acid metabolism, glucose transportation, and growth performance (18, 19). L-isoleucine can improve the immune system, including immune organs, cells, and reactive substances (20). According to our findings, L-isoleucine was upregulated (4.714-fold) and 14 different amino acids were downregulated in UV-WSSV trained immunity shrimp, suggesting that UV-WSSV training enhanced protein metabolism in shrimp.

#### Identification of DMs in EW vs. EU Groups

In EW vs. EU groups (**Table 3**), 23 DMs were identified: 11 were upregulated and 12 were downregulated. Quinic acid was upregulated by 5-fold and urea by approximately 2-fold, while the most downregulated metabolites included L-iditol, uridine, cholesterol, beta-D-glucose-6-phosphate, beta-D-fructose-6-phosphate, and D-fructose-6-phosphate. Based on *p* values and VIP scores, D-fructose-6-phosphate, succinate, glycylglycine, fumaric acid, and urea were the most significantly altered metabolites; of them, glycylglycine, succinate, and fumaric were upregulated. As evident from **Supplementary Figures 3C, D**, succinate and fumaric acid were significantly upregulated in the EW group. Further, beta-D-glucose, beta-D-glucose-6-phosphate, and D-fructose-6-phosphate, which are all associated with the glycolysis pathway, were significantly downregulated in the EW group. These results indicated that the glycolysis pathway might be involved in UV-WSSV-induced trained immunity in shrimp.

### Heatmaps of DMs

**Figure 5:**
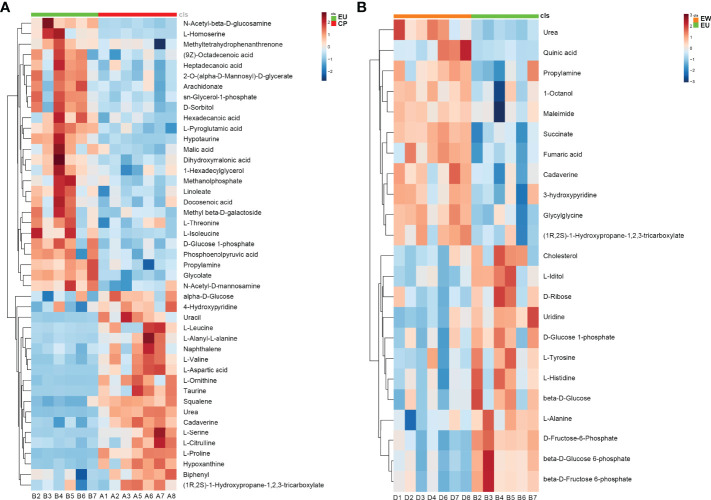
Heatmaps plotted using significantly changed metabolites. Each metabolite is represented by a row of colored boxes. Columns represent samples, and rows represent metabolites. Red indicates an increase in the relative level of metabolites, while blue indicates a decrease. **(A)** Heatmap of EU vs. CP and **(B)** EW vs. EU groups. A = CP, B = EU, and D = EW.

We performed hierarchical clustering analyses to assess metabolic patterns under different experimental conditions; relative changes in various metabolites were determined and heatmaps were plotted (**Figure 5**). We found that the levels of metabolite change of six biological duplications within the groups showed good reproducibility, suggesting that our results were highly reliable. We could more intuitively identify downregulated metabolites, such as urea, L-proline, taurine, and L-aspartic acid, as well as upregulated metabolites, including L-isoleucine, L-homoserine, glycolate, and propylamine, in EU vs. CP groups (**Figure 5A**). Metabolites including quinic acid, maleimide, succinate, and fumaric acid were upregulated in EW vs. EU groups and those such as cholesterol, beta-D-glucose-6-phosphate, beta-D-fructose-6-phosphate, and L-iditol were downregulated (**Figure 5B**). All findings suggested that metabolism was significantly altered in EU vs. CP and EW vs. EU groups and that metabolic pathways were distinct between these pairs of groups (reprogramed).

### Comparison of DMs Between Experimental and Control Groups

**Figure 6:**
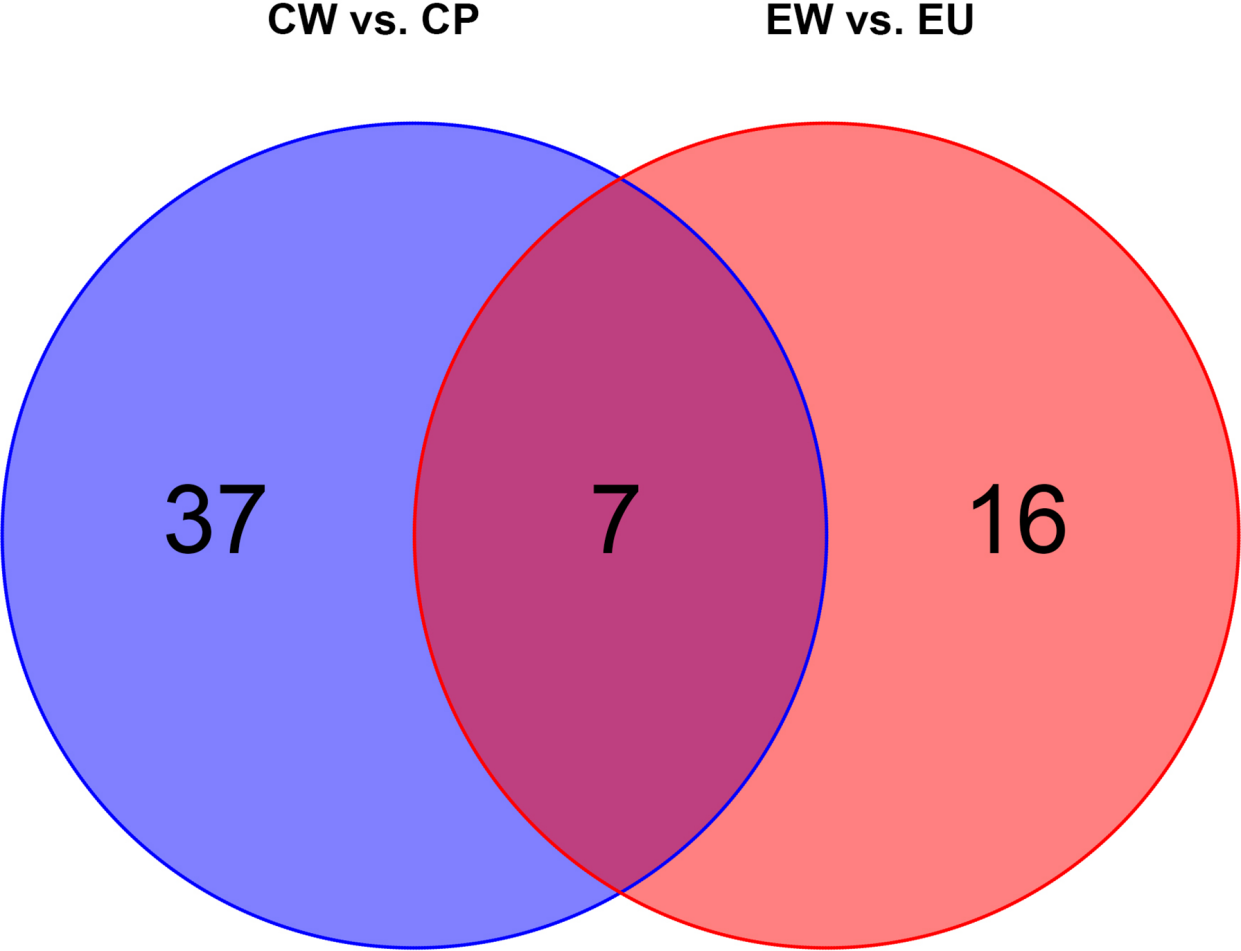
Venn diagram showing common and unique metabolites (EW vs. EU and CW vs. CP groups). There are 16 and 37 unique differential metabolites in EW vs. EU and CW vs. CP groups, respectively. Seven differential metabolites were common between them.

We plotted a Venn diagram (**Figure 6**) to determine the number of common and unique DMs between experimental and control groups. As anticipated, there were certain changes in metabolites between EW vs. EU and CW vs. CP groups. Detailed information pertaining to DMs is shown in **Table 4**. In the experimental group (EW vs. EU), only 16 DMs were identified, including D-fructose-6-phosphate, beta-D-glucose-6-phosphate, and beta-D-fructose-6-phosphate, whereas in the control group (CW vs. CP), 37 unique DMs were identified, including squalene, L-proline, and L-valine. Seven DMs were common between the groups (**Figure 6**). These results suggested that UV-WSSV training led to metabolism reprogramming in UV-WSSV trained shrimp. In other words, trained immunity showed a relationship with metabolism in shrimp.

**Table 4:** Differential metabolites in the Venn diagram of EW vs. EU and CW vs. CP *groups*.

Compound	CW vs. CP	EW vs. EU
2,6-ditert-butylphenol	Y	N
Squalene	Y	N
L-Proline	Y	N
Hypotaurine	Y	N
1,5-Anhydro-D-glucitol	Y	N
N-Acetyl-D-mannosamine	Y	N
Linoleate	Y	N
Hexadecanoic acid	Y	N
L-Valine	Y	N
N-Acetyl-beta-D-glucosamine	Y	N
L-Ornithine	Y	N
Hypoxanthine	Y	N
L-Pyroglutamic acid	Y	N
Heptanoic acid	Y	N
L-Threonine	Y	N
L-Aspartic acid	Y	N
Taurine	Y	N
Uracil	Y	N
6-Aminohexanoate	Y	N
L-Serine	Y	N
Cellobiose	Y	N
Phosphoenolpyruvic acid	Y	N
1-Hexadecylglycerol	Y	N
L-Alanyl-L-alanine	Y	N
L-Citrulline	Y	N
Sucrose	Y	N
11-Eicosenoic acid	Y	N
2-O-(alpha-D-Mannosyl)-D-glycerate	Y	N
Malic acid	Y	N
Naphthalene	Y	N
Uridine-5-diphospho-N-acetylglucosamine	Y	N
L-Leucine	Y	N
Ribitol	Y	N
N-Acetylserotonin	Y	N
Heptadecanoic acid	Y	N
Guanosine	Y	N
Nonanoic acid	Y	N
D-Fructose-6-Phosphate	N	Y
Glycylglycine	N	Y
Uridine	N	Y
beta-D-Glucose-6-phosphate	N	Y
beta-D-Fructose-6-phosphate	N	Y
L-Iditol	N	Y
(1R,2S)-1-Hydroxypropane-1,2,3-tricarboxylate	N	Y
L-Alanine	N	Y
D-Ribose	N	Y
L-Tyrosine	N	Y
1-Octanol	N	Y
L-Histidine	N	Y
Quinic acid	N	Y
Maleimide	N	Y
beta-D-Glucose	N	Y
Cholesterol	N	Y
Fumaric acid	Y	Y
Urea	Y	Y
3-Hydroxypyridine	Y	Y
Propylamine	Y	Y
Cadaverine	Y	Y
Succinate	Y	Y
D-Glucose-1-phosphate	Y	Y

Y, DM present; N, DM absent.

On plotting a Venn diagram, we found similarities and differences in metabolites between experimental (EU vs. EW) and control (CP vs. CW) groups. Energy metabolism-related metabolites, fumaric acid, and succinate were upregulated in both the groups, but urea was significantly upregulated in the experimental group (EW vs. EU) and downregulated in the control group (CW vs. CP). Many amino acids were altered in CW vs. CP groups. L-Proline, L-Valine, L-Ornithine, L-Aspartic acid, Taurine, L-Serine, L-Alanyl-L-alanine, L-Citrulline and L-Leucine were exclusively altered in CW vs. CP groups, which were down-regulated. Hypotaurine, L-Pyroglutamic acid, L-Threonine were exclusively altered in CW vs. CP groups, which were up-regulated. D-Fructose-6-Phosphate, beta-D-Glucose-6-phosphate, beta-D-Fructose-6-phosphate, D-Ribose, 1-Octanol, Quinic acid, beta-D-Glucose and cholesterol were unique in EW vs. EU. Most unique metabolites in EW vs. EU groups were related to energy metabolism. Cholesterol only changed in the trained group, to which is worth paying attention.

### Metabolic Pathway Analyses

**Figure 7:**
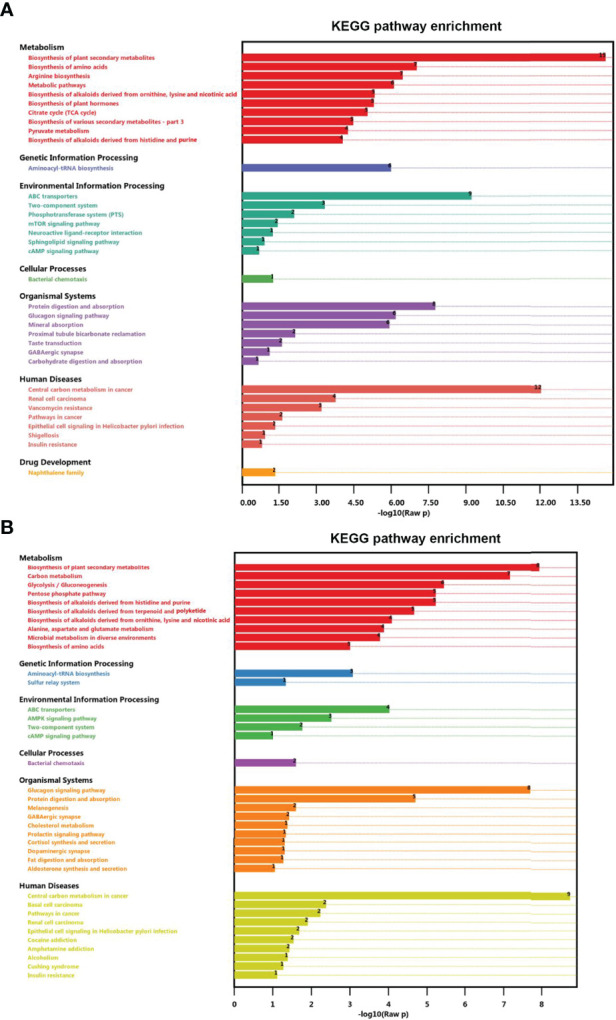
Enrichment of differential metabolites in distinct KEGG pathways. **(A)** CP vs. CW and **(B)** EU vs. EW groups.

To explore potential metabolic pathways affected by UV-WSSV training, all DMs were annotated by KEGG pathway analysis. In the control group (CP vs. CW), biosynthesis of amino acid, biosynthesis of unsaturated fatty acid, TCA cycle, and mTOR signaling pathway were prevalent (**Figure 7A**), whereas in the experimental group EW vs. EU, carbon metabolism, glycolysis/gluconeogenesis, pentose phosphate pathway, and AMPK signaling pathway were enriched (**Figure 7B**).

### Methylation of Histones at H3K4 Was Involved in Trained Immunity

**Figure 8:**
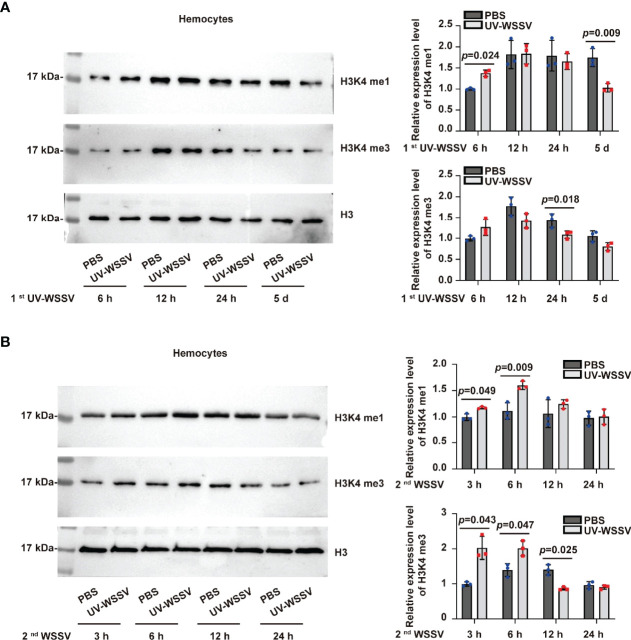
H3K4me1 and H3K4me3 were involved in trained immunity in shrimp. Histones were extracted from PBS and UV-WSSV trained shrimp at different time points, and H3K4me1 and H3K4me3 were analyzed using western blotting. **(A)** H3K4me1 modification analyzed by western blotting using anti-H3K4me1 as the primary antibody and statistical analysis based on three experiments. **(B)** H3K4me3 modification analysis using anti-H3K4me3 as the primary antibody and statistical analysis based on three experiments. The bands of western blotting were digitalized using Image J software, and the differences between trained and control groups at different time points was analyzed by student’s *t* test, *p* < 0.05 was accepted as significant difference.

Fumaric acid was significantly upregulated in shrimp at the 2^nd^ WSSV infection as compared to those with 1^st^ UV-WSSV training (**Table 3**). A previous study reported that fumarate-induced trained immunity resulted in increased trimethylation of histone at H3K4 (H3K4me3), associating metabolic activation with epigenetic changes (15). In the experimental (EU and EW) groups, we found that fumaric acid was upregulated (**Table 3**), indicating that histone methylation modification might be involved in trained immunity in shrimp. We therefore analyzed methylation of histones at H3K4, including monomethylation of histone H3 at lysine 4 (H3K4me1) and H3K4me3. The results showed that during training period, both H3K4me1 and H3K4me3 were increased at 6 h post UV-WSSV injection compared to PBS injection group and recovered to basal level at the 5th day of training (**Figure 8A**). After the 2^nd^ infection with WSSV, the H3K4me1 and H3K4me3 was increased significantly at 3 h compared to PBS injection group (**Figure 8B**), suggesting a faster and stronger modification in UV-WSSSV trained shrimp. All the results suggested that modification of H3K4me1 and H3K4me3 is involved in the trained immunity in shrimp.

## Discussion

In the present study, UV-WSSV was used to induce trained immunity in *M. japonicus* and metabolic changes were analyzed. Metabolomic analyses revealed distinct changes between various groups. These changes were associated with several trained immunity-related metabolic pathways.

The ability of the innate immune system to develop adaptive characteristics (enhanced innate immune response to pathogens after an initial challenge) and provide long-term protection against pathogenic reinfection (*via* innate immune memory) is termed as trained immunity (21). However, there are several similarities between the trained immunity and other immune processes, such as differentiation, priming and tolerance. The main difference of these adaptive programs in innate immune cells is their functional status prior to secondary challenges (10). For the innate immune cell differentiation, cells are not allowed to return to the functional steady state before secondary stimulation. In the priming process, the first stimulus changes the functional state of cells, and their immune status does not return to basal levels before the secondary stimulation or infection (3). The tolerance is opposite process with trained immunity, innate immune cell is unable to activate gene transcription and does not perform its functions following second stimulation (10). The most common model of trained immunity is the training of innate immune cells or organisms for a short duration (10). Subsequently, cells or organisms rest for 5–7 days without any stimulation, and functional programs of cells return to a low and steady state during the resting phase (10). On the second stimulation, a heightened response to homologous or heterologous secondary stimuli is induced (10). In shrimp, the resting period after pathogen priming should be at least 5 days for the low recovery of immune response level (7). Considering this, we chose 5 days as the resting period after UV-WSSV training. Furthermore, the survival rate of the UV-WSSV trained immunity group was significantly higher than that of the control group. Vago-L inhibits white spot syndrome virus infection by activating JAK/STAT signaling in shrimp (22). Ficolin is an antiviral effector through binding virions and promoting phagocytosis, which is a direct antiviral effector downstream the WSSV/Vago-L/JAK/STAT axis in *M. japonicus* (22). We investigated the expression patterns of *Vago-L* and *Ficolin* in *M. japonicus*, and the results showed that expression of the two genes showed faster and stronger gene expressions after the second challenge with WSSV in UV-WSSV training shrimp (**Figures 1F, G**), which is consistent with the important characteristics of trained immunity (10). By methylation analysis, we found that H3K4me1 and H3K4me3 modification recovered to basal level at 5th day of UV-WSSV stimulation and the level of modifications increased faster and greater compared to control group (**Figure 8**). All the results suggested that UV-WSSV treatment induced trained immunity, and we successfully established the model of trained immunity in *M. japonicus*.

On comparing EU vs. CP groups, 45 DMs were identified, indicating that UV-WSSV treatment induced metabolome reprogramming in *M. japonicus*. The highest number of DMs were involved in “metabolic pathways” and “biosynthesis of amino acids.” As mentioned earlier, a lot of differential amino acids were identified, with most being downregulated in EU group. In addition, six metabolites that were the most up-regulated (L-isoleucine, L-homoserine, and hypotaurine) and downregulated (L-proline, taurine, and L-aspartic acid) from a fold-change perspective were amino acids. These results suggested that UV-WSSV training affected protein synthesis and amino acid metabolism. Amino acid metabolism plays a key role in metabolic rewiring and supports diverse immune cell functions (23). In addition, amino acids contribute to many other intracellular metabolic pathways. Immune cells rely on these pathways to obtain energy and biomass, and reprogram their metabolism after activation to support growth, proliferation, and effector functions (23). Accordingly, our findings suggested that amino acid metabolism and protein synthesis were involved in UV-WSSV-induced trained immunity. L-proline reportedly increases DNA methylation to regulate the pluripotency of embryonic stem cells (24). And synthesized cadmium–proline complexes accelerate epigenetic rearrangement by histone deacetylases inhibition (25). The treatment of cultured hepatocellular carcinoma cells with hydrogen peroxide caused methylation of the E-cadherin promoter (26). It showed that the ROS generated by proline degradation induces the expression of a genetic program including antioxidant enzymes protecting against ROS so that lifespan is extended (27, 28). Epigenetic reprogramming is a widely recognized trained immunity mechanism, and it mainly involves changes in chromatin structure caused by histone modifications, such as methylation and acetylation (29). Our data suggests that L-proline is involved in this process, affecting epigenetic modifications *via* metabolic changes and thereby inducing UV-WSSV trained immunity in *M. japonicus*. During amino acid metabolism *in vivo*, ammonia is produced through ornithine, followed by urea synthesis; this process is known as the ornithine cycle or the urea cycle. As highly concentrated ammonia is toxic to cells, most of it is converted to urea through the ornithine cycle. This might explain the high folds change of urea among all DMs in different groups.

It is reported that training of monocytes with β-glucan induces profound changes in cellular metabolism. Three metabolic pathways are involved in trained immunity, namely glycolysis, glutaminolysis, and cholesterol synthesis, which are linked to enrichment in H3K4me3 (15). mTOR signaling pathway is complicated and is differently regulated under different conditions. A study reported that inhibition of Akt, mTOR, or HIF-1α blocked the trained immunity induced by the *Candida albicans* cell wall constituent β-glucan in human monocytes (14). We herein observed that the mTOR signaling pathway was significantly altered, with L-leucine being significantly downregulated within this pathway. These results indicated that the changes in amino acid metabolism were particularly significant in this process.

Energy metabolism is one of the driving forces for the performance of immunity. Several DMs were enriched in the energy metabolism pathway in EU vs. CP groups. Arachidonate, a long-chain polyunsaturated fatty acid, has beneficial effects on the immunity of aquatic animals (30, 31), which was upregulated in our result. It is related with linoleic acid metabolism. In our previous study, we found that linoleic acid plays a key role against WSSV infection (32). Therefore, its upregulation might be related to trained immunity.

In EW vs. EU groups, there were 23 DMs, including succinate (**Table 3**). Succinate is recently being receiving increased attention as an epigenetic modulator, and it is also involved in reactive oxygen species (ROS) formation and elimination, signal transduction, and endo- and paracrine modulation and inflammation (33). β-glucan training has been reported to enhance ROS levels in tumor-associated neutrophils, and ROS production is the basic feature of trained neutrophils to exert anti-tumor activity (34). Our findings indicated that succinate was significantly upregulated, suggesting that it participates in UV-WSSV-induced trained immunity. Succinate is oxidized by succinate dehydrogenase to fumaric acid to participate in the TCA cycle. The TCA cycle is the most effective way to oxidize sugars or other substances so as to generate energy. Further, TCA cycle is the final metabolic pathway of carbohydrates, lipids, as well as amino acids, and it is the hub of their metabolic connection. This explains why fumaric acid was also significantly upregulated herein. Fumarate-induced trained immunity results in increased H3K4me3 and also acetylation at H3K27; it is notable that fumarate accumulation integrates immune and metabolic circuits to induce monocyte epigenetic reprogramming by inhibiting KDM5 histone demethylases (15). We observed that fumaric acid was significantly upregulated in EW vs. EU groups, and H3K4me1 and H3K4me3 were increased in UV-WSSV trained immunity shrimp. Long-term changes in DNA methylation and stable changes in chromatin accessibility can accompany cell differentiation, whereas specific histone marks characterizing ‘latent enhancers,’ such as monomethylated histone H3K4 are often ‘tagged’ in trained immunity (6, 21). In the present study, we also found that H3K4me1 and H3K4me3 were involved in the UV-WSSV trained immunity.

Energy is indispensable for various life activities, including immunity. We found that beta-D-glucose-6-phosphate, beta-D-fructose-6-phosphate, and D-fructose-6-phosphate showed an obvious downregulation (**Table 3**), indicating that energy metabolism was affected. In addition, KEGG pathway analysis revealed the enrichment of glucagon signaling pathway and glycolysis, suggesting an increase in energy consumption in shrimp with trained immunity. After trained immunity, the immune response to homologous infection is evidently stronger (2, 4); thus, we speculated that higher energy metabolism must be related to this response. Monocytes/macrophages appear to have a central role in COVID-19 pathogenicity; they adapt their metabolism upon infection and become highly glycolytic, facilitating SARS-CoV-2 replication (35). The infection triggers mitochondrial ROS production, which induces stabilization of hypoxia-inducible factor-1α and consequently promotes glycolysis (35). Similarly, WSSV infection has been found to increase energy demand and induce the Warburg effect, in which glucose consumption and lactate production increase even in the presence of oxygen, and this is essential for viral replication (36, 37). We observed that shrimp with UV-WSSV trained immunity showed increased energy metabolism after the second injection with WSSV.

Bekkering et al. (38) reported that monocytes from patients with familial hypercholesterolemia showed a trained immunity phenotype and that lipid lowering with statins did not revert this proinflammatory phenotype. Moreover, Wang et al. (39) reported that an *M. japonicus* stomach virus-associated C-type lectin (MjsvCL) enhanced WSSV entry *via* the MjsvCL–calreticulin pathway in a cholesterol-dependent manner. We found a decrease in cholesterol levels on comparing EW vs. EU groups. Accordingly, we believe that the decrease in cholesterol was a strategy against WSSV infection in shrimp trained immunity.

Interestingly, we found that quinic acid level was significantly upregulated in EW vs. EU groups. Quinic acid, a cyclohexanecarboxylic acid, is generally obtained from plants (40), and it has been mostly studied as a chlorogenic acid, an ester of caffeic and quinic acids (41). In the colon, chlorogenic acid is hydrolyzed by microbial esterases to release caffeic and quinic acids (41). Quinic acid derivatives have been found in propolis produced by *Apis mellifera* in the South and Southeast regions of Brazil (42). Furthermore, quinic acid derivatives evidently exert antiviral activities against HIV, Moloney murine leukemia virus, and dengue virus (43, 44). The hemolymph of crustaceans has been reported to have microbiota (45, 46). Quinic acid levels were found to be positively correlated with the abundance of some probiotic bacteria such as *Lactobacillus* in the gut, with changes in gene modulation effects (47, 48). Two hundred and two strains of lactic acid bacteria isolated from digestive tracts of cultivated and wild adult shrimp, including *Litopenaeus vannamei*, *Metapenaeus brevicornis* and *Penaeus merguiensis* (49). In addition, *Lactobacillus* species as probiotics was used in shrimp aquaculture to prevent viral infections due to their positive promoting effects on survival and health (50). It is reasonable to assume that quinic acid plays a role against WSSV; nevertheless, further studies are warranted to validate its function.

To determine metabolic changes underlying UV-WSSV-induced trained immunity in shrimp, we performed metabolomic-based analyses, which led to the identification of several DMs, many of which were closely related to amino acid metabolism. Further, we assessed changes in the EW group in comparison with the EU group and found that metabolites related to energy and amino acid metabolism, including succinate, fumaric acid, and urea, were the most significantly altered. Many energy metabolism-related DMs were found only in the trained immunity groups, while more amino acid metabolism-related DMs were found in the control groups. We believe that glycolysis, pentose phosphate pathway, and AMPK signaling pathway are involved in WSSV-induced trained immunity in shrimp and that metabolic changes during the process of trained immunity are associated H3K4me1 and H3K4me3 epigenetic modifications.

## Materials and Methods

### Animals and Rearing Conditions

Healthy shrimp (*M. japonicus*) with average body weight of 20 ± 2 g were obtained from a local farming pond in Aoshanwei, Qingdao, China. They were maintained in aerated natural seawater at 20°C in our laboratory and acclimated for 1 day before the training experiment. Animals were randomly selected for subsequent experiments. The animal treatment protocol was reviewed and approved by Shandong University School of Life Sciences.

### Preparation of Inactivated Viral Inoculum

WSSV inoculum was prepared as previously described, and qRT-PCR was performed for viral quantification (22, 51).

WSSV was inactivated by exposure to UV (UV-WSSV). Briefly, WSSV inoculum (15 mL, 1 × 10^7^ copies/mL) in a sterile Petri dish was placed under a 20 W UV lamp on an ultraclean workbench for 30 min. The irradiation distance was 500 mm. Before the training experiment, we performed shrimp mortality analysis to detect the training effect of UV-WSSV. The number of dead shrimp was counted every 24 h, and the survival rate of shrimp was calculated using the log-rank (Mantel–Cox) test.

### UV-WSSV-Induced Trained Immunity Model

**Figure 1A** shows the establishment of the UV-WSSV-induced trained immunity model in shrimp. Shrimp were randomly divided into two groups (100 shrimp/group). Fifty microliters of UV-WSSV (5 × 10^5^ copies) were intramuscularly injected into the animals. The control group was injected with the same volume of sterile PBS (140 mM NaCl, 2.7 mM KCl, 10 mM Na_2_HPO_4_, and 1.8 mM KH_2_PO_4_, pH 7.4). Five days after UV-WSSV training, a second injection of WSSV was administered. The training and control groups were injected with the same amount of live WSSV (50 μL, 5 × 10^5^ copies).

### Detection of Viral Replication

After WSSV infection, the hemocytes of trained shrimp (UV-WSSV/PBS) was sampled to isolated total RNA at 12 and 24 h, and qRT-PCR was performed with the reverse transcribed RNA samples (22, 51) to detect the *Vp28* expression level.

Prior to treatment, the shrimp used for trained immunity analysis were randomly selected for testing WSSV infection by RT-PCR with primers VP28F and VP28R (**Supplementary Table 3**). Hemocytes, intestine and gills of untreated shrimp were sampled to isolated total RNA with TRIzol (Transgen, Beijing, China), and RT-PCR was carried out to detect the *Vp28* expression level with the reverse transcribed RNA. The PCR procedure comprised: an initial incubation at 94°C for 3 min; followed by 28 cycles of 94°C for 30 s, 60°C for 30 s, and 72°C for 30 s; followed by 72°C for 10 min. The PCR products were analyzed by agarose gel electrophoresis (1.2% agarose). ddH2O was used as a negative control, while the WSSV sample was used as a positive control.

### qRT-PCR

The total RNA was isolated from the hemocytes of the PBS- or UV-WSSV-treatment shrimp at 6, 12, 24 h and 5 days post injection. After second challenge with WSSV, the hemocytes was sampled to isolated total RNA at 3, 6, 12 and 24 h post infection. Then qRT-PCR was performed with the reverse transcribed RNA samples (22, 51).

β-Actin was used as a control. Each assay was carried out in triplicate with the following cycling conditions: 94°C for 10 min, followed by 40 cycles at 94°C for 15 s, and 62°C for 50 s; and then a melting period from 65°C to 95°C (22, 51). The gene relative expression levels of *Vago-L* and *Ficolin* were detected. Triplicate Ct values were analyzed by the comparative Ct (ΔΔCt) method. The data are presented as mean ± standard deviation (SD). The primers are listed in **Supplementary Table 3**.

### Metabolomic Sample Collection

For metabolome analyses, hemolymph was sampled at two timepoints: first in the training phase and another after active WSSV reinjection (**Figure 1A**). At 48 h of training, hemolymph samples from both groups (experiment/control) were collected [hemolymph from the control group (CP, control group injected with PBS) and from the UV-WSSV trained immunity group (EU, experimental group injected with UV-WSSV)]. Subsequently, at 24 h of the second injection with WSSV, hemolymph samples were again collected from both groups [hemolymph from the control and UV-WSSV trained immunity groups injected with WSSV (CW and EW, respectively), **Figure 1A**)].

Hemolymph samples were collected from two shrimp using a syringe preloaded with 500 μL anticoagulant (450 mM NaCl, 10 mM KCl, 10 mM EDTA, and 10 mM HEPES, pH 7.45) at a ratio of 1:1, gently mixed, and clearly marked. The samples were then centrifuged (4°C, 1600 ×*g*, 15 min) as soon as possible and the supernatant (plasma) thus obtained was immediately frozen in liquid nitrogen. All samples were stored at −80°C until needed. In total, for each group, >6 biological duplicate samples were subjected to metabolomic analyses.

### Metabolomic Analysis

At least six plasma samples from each group were assessed using tandem gas chromatography mass spectrometry (GC–MS/MS) by BioNovoGene company (Suzhou, China). Briefly, 1 mL ice-cold (−20°C) acetonitrile:isopropanol:water (3:3:2, v/v/v) was added to 50 μL sample and vortexed for 30 s. After ultrasonication for 5 min at room temperature, the samples were centrifuged at 13523 ×*g* for 2 min. Subsequently, 500 μL of the supernatant was dried in a vacuum concentrator for 8–10 h. The sample was then redissolved in 80 μL of 20 mg/mL methoxypyridine and thoroughly mixed for 30 s, followed by incubation at 60°C for 1 h. Finally, 100 μL BSTFA-TMCS (99:1) reagent was added to the samples, vortexed for 30 s, followed by heating at 70°C for 90 min. After centrifugation at 18407 ×*g* for 3 min, the supernatant (100 μL) was added to the detection bottle. Overall samples were placed in sealed cuvettes stored for testing and processed for gas chromatography time-of-flight upper detection within 24 h (52).

Gas chromatographic separations of derivatives were performed on a DB-5MS capillary column (30 m × 250 μm i.d., 0.25 μm film thickness, Agilent J & W Scientific, Folsom, CA, USA) with a constant flow of 1 mL/min helium. The sample (1 μL) was injected in split mode at a split ratio of 1:10 by the autosampler. The inlet temperature was 280°C, and the temperatures of the transfer line and ion source were 320°C and 230°C, respectively. The column temperature was initially held at 50°C for 30 s and then increased at a rate of 15°C/min to 320°C; the temperature was then maintained at 320°C for 9 min. MS was performed using the full-scan method at a scan rate of 10 spectra/s, electron energy of −70 V, and solvent delay of 3 min (53, 54).

First, all sample data were preprocessed. A data matrix was obtained, including mass-to-charge ratio (*m*/*z*), retention time (rt), and intensity. The minimum one-half method to fill in the blanks was used to simulate the missing value recoding of the original data. To compare different magnitudes, data were subjected to total peak area normalization. All data were performed quality assurance and quality control (55). Data were normalized using autoscaling (mean-centering and scaled to unit variance) before performing multivariate analysis, including PCA, and OPLS-DA to obtain more reliable, intuitive results. SIMCA-P v13.0 and R package ‘ropls’ (56) were used. Based on *p* ≤ 0.05 and VIP score ≥ 1 obtained by OPLS-DA (57), DMs were identified. The R v3.3.2 package pheatmap was used to zoom the dataset and to obtain a hierarchical clustering map of relative quantitative values of metabolites. DMs were annotated by performing KEGG pathway analysis using MetPA, which is a part of metaboanalyst (www.metaboanalyst.ca) and mainly based on KEGG metabolic pathways.

### Histone Modification Analysis

Histones were extracted using the Nuclear Protein Extraction Kit (R0050, Solarbio, Beijing, China), according to manufacturer instructions. Further, we analyzed histone modification levels by western blotting (58) using anti-H3K4me1 and anti-H3K4me3 as primary antibodies (ABclonal, USA). Histone H3 polyclonal antibody (ABclonal, USA) was used as the reference. Student’s *t* test statistical analysis of H3K4me1 and H3K4me3 modifications were performed after digitalized the bands of western blotting using Image J software.

### Statistical Analysis

Values are presented as mean ± SD. *p* < 0.05 indicated statistically significant differences analyzed by Student’s *t* test.

## Data Availability Statement

The original contributions presented in the study are included in the article/**Supplementary Material**. Further inquiries can be directed to the corresponding author.

## Author Contributions

SZ performed experiments, including establishment of the shrimp trained immunity model, sample collection, performed the metabolomics analysis, and wrote and revised the manuscript draft, and helped to design the study. L-XL and C-FL contributed to the model establishment and samples collection. L-XL and PZ performed histone modification analysis and expression patterns of immune genes. C-FL and CL prepared the WSSV inoculum for infection. J-XW designed and administrated this study, wrote and revised the manuscript. All authors contributed to the article and approved the submitted version.

## Funding

This work was supported by grants from the National Natural Science Foundation of China (Grant Nos. 31930112), National Key Research and Development Program of China (Grant No. 2018YFD0900502).

## Conflict of Interest

The authors declare that the research was conducted in the absence of any commercial or financial relationships that could be construed as a potential conflict of interest.

## Publisher’s Note

All claims expressed in this article are solely those of the authors and do not necessarily represent those of their affiliated organizations, or those of the publisher, the editors and the reviewers. Any product that may be evaluated in this article, or claim that may be made by its manufacturer, is not guaranteed or endorsed by the publisher.
