# Supplementary material for: Metabolomic Investigation of Ultraviolet Ray-Inactivated White Spot Syndrome Virus-Induced Trained Immunity in Marsupenaeus japonicus
Source: Front Immunol. 2022 May 26;13:885782. doi: 10.3389/fimmu.2022.885782 (PMC9178177; doi:10.3389/fimmu.2022.885782)
Supplement: Supplementary file 1 [file DataSheet_1.pdf]

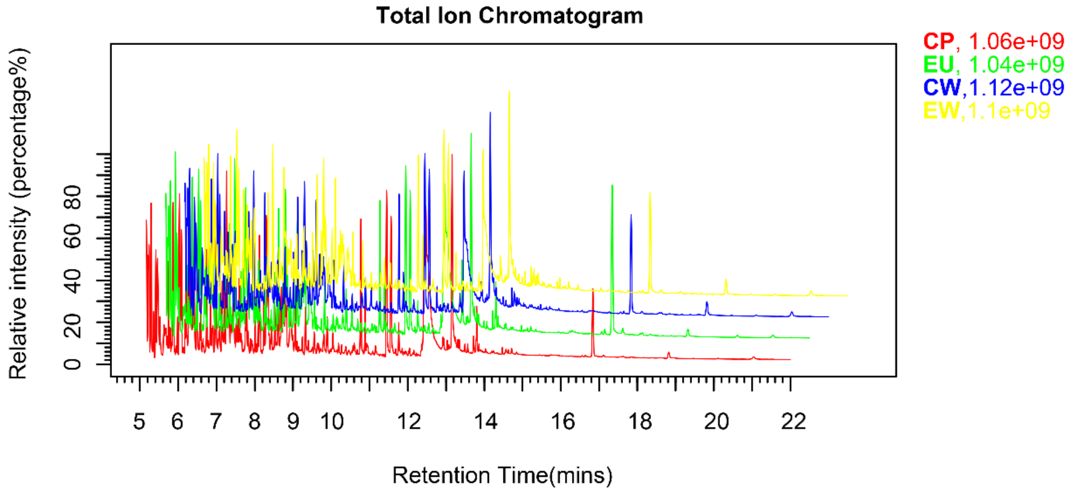

Supplementary Figure 1. Total ion chromatogram of *M. japonicus* plasma samples from the four groups using GC–MS/MS
