# Supplementary material for: Metabolomic Investigation of Ultraviolet Ray-Inactivated White Spot Syndrome Virus-Induced Trained Immunity in Marsupenaeus japonicus
Source: Front Immunol. 2022 May 26;13:885782. doi: 10.3389/fimmu.2022.885782 (PMC9178177; doi:10.3389/fimmu.2022.885782)
Supplement: Supplementary file 3 [file DataSheet_3.pdf]

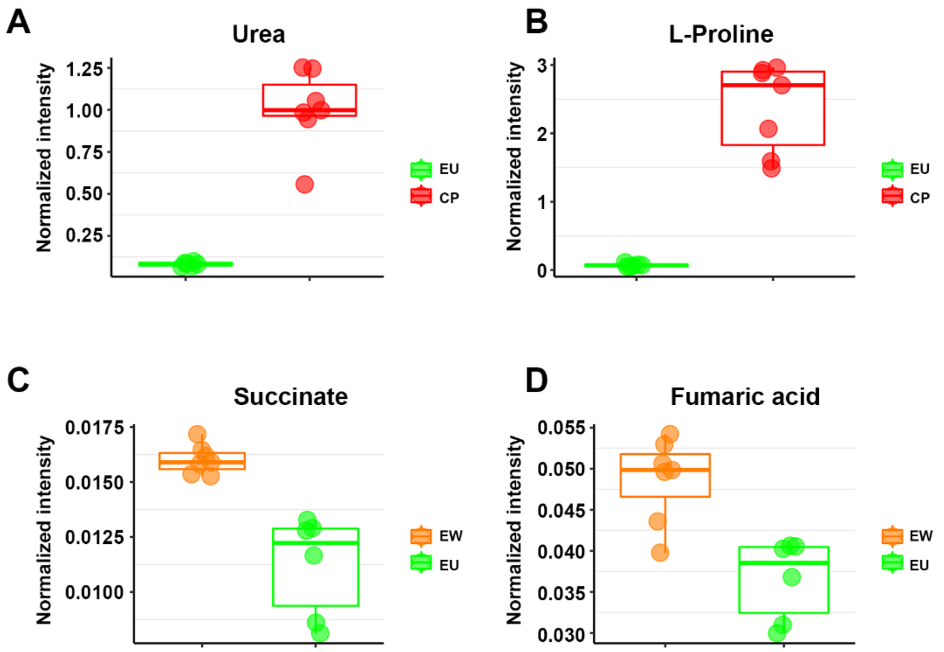

Supplementary Figure 3. Data dispersion of **(A)** urea and **(B)** L-proline in EU vs. CP groups. Data dispersion of **(C)** succinate and **(D)** fumaric acid in EW vs. EU groups.
