# Supplementary material for: Metabolomic Investigation of Ultraviolet Ray-Inactivated White Spot Syndrome Virus-Induced Trained Immunity in Marsupenaeus japonicus
Source: Front Immunol. 2022 May 26;13:885782. doi: 10.3389/fimmu.2022.885782 (PMC9178177; doi:10.3389/fimmu.2022.885782)
Supplement: Supplementary file 4 [file Table_1.docx]

Supplementary Table 1

Significantly changed differential metabolites in CW vs. CP groups

| Compound | Fold change | *p*-value | VIP |
| --- | --- | --- | --- |
| 1,5-Anhydro-D-glucitol | 5.418 | 4.49E-05 | 1.804 |
| Hypotaurine | 4.135 | 7.10E-06 | 1.869 |
| L-Threonine | 2.628 | 0.0011 | 1.615 |
| N-Acetyl-beta-D-glucosamine | 2.499 | 0.000235 | 1.722 |
| Sucrose | 1.927 | 0.0138 | 1.341 |
| Fumaric acid | 1.764 | 2.06E-06 | 1.902 |
| Phosphoenolpyruvic acid | 1.665 | 0.0104 | 1.379 |
| Propylamine | 1.651 | 0.000703 | 1.65 |
| Succinate | 1.626 | 0.0139 | 1.339 |
| 1-Hexadecylglycerol | 1.625 | 0.0113 | 1.369 |
| Ribitol | 1.598 | 0.022 | 1.269 |
| 2,6-ditert-butylphenol | 1.568 | 6.97E-09 | 1.983 |
| 2-O-(alpha-D-Mannosyl)-D-glycerate | 1.531 | 0.0157 | 1.322 |
| Uridine-5-diphospho-N-acetylglucosamine | 1.43 | 0.0179 | 1.302 |
| 11-Eicosenoic acid | 1.314 | 0.0155 | 1.323 |
| Cellobiose | 1.309 | 0.00605 | 1.448 |
| L-Pyroglutamic acid | 1.304 | 0.000349 | 1.698 |
| N-Acetylserotonin | 1.299 | 0.0253 | 1.246 |
| Linoleate | 1.257 | 0.000193 | 1.733 |
| N-Acetyl-D-mannosamine | 1.243 | 0.000126 | 1.756 |
| Heptanoic acid | 1.241 | 0.00038 | 1.692 |
| Heptadecanoic acid | 1.231 | 0.0366 | 1.181 |
| D-Glucose-1-phosphate | 1.219 | 0.0317 | 1.207 |
| Malic acid | 1.196 | 0.0165 | 1.314 |
| 3-Hydroxypyridine | 1.103 | 0.00016 | 1.744 |
| 6-Aminohexanoate | 1.091 | 0.00354 | 1.508 |
| Hexadecanoic acid | 1.06 | 0.000212 | 1.728 |
| Nonanoic acid | 0.884 | 0.0498 | 1.121 |
| Cadaverine | 0.621 | 0.00328 | 1.516 |
| Naphthalene | 0.614 | 0.0177 | 1.304 |
| Guanosine | 0.304 | 0.0447 | 1.143 |
| L-Citrulline | 0.252 | 0.0126 | 1.353 |
| Urea | 0.245 | 7.16E-05 | 1.784 |
| L-Alanyl-L-alanine | 0.227 | 0.0114 | 1.368 |
| Squalene | 0.202 | 1.23E-08 | 1.978 |
| Uracil | 0.175 | 0.00217 | 1.556 |
| L-Valine | 0.145 | 0.000219 | 1.726 |
| Hypoxanthine | 0.122 | 0.00031 | 1.705 |
| L-Ornithine | 0.086 | 0.000264 | 1.715 |
| L-Serine | 0.068 | 0.00373 | 1.502 |
| L-Proline | 0.039 | 3.33E-06 | 1.89 |
| L-Leucine | 0.038 | 0.0206 | 1.28 |
| L-Aspartic acid | 0.024 | 0.00132 | 1.6 |
| Taurine | 0.021 | 0.00185 | 1.571 |
