# Supplementary material for: Metabolomic Investigation of Ultraviolet Ray-Inactivated White Spot Syndrome Virus-Induced Trained Immunity in Marsupenaeus japonicus
Source: Front Immunol. 2022 May 26;13:885782. doi: 10.3389/fimmu.2022.885782 (PMC9178177; doi:10.3389/fimmu.2022.885782)
Supplement: Supplementary file 5 [file Table_2.docx]

Supplementary Table 2

Significantly changed differential metabolites in EW vs. CW groups

| Compound | Fold change | *p*-value | VIP |
| --- | --- | --- | --- |
| Quinic acid | 8.405 | 0.0339 | 1.659 |
| Propylamine | 1.161 | 0.00106 | 2.247 |
| Succinate | 1.157 | 0.0073 | 1.979 |
| Tetradecanoic acid | 1.122 | 0.0438 | 1.592 |
| 1-Octanol | 1.121 | 0.00103 | 2.25 |
| Maleimide | 1.069 | 0.0414 | 1.607 |
| 9-Hexadecenoic acid | 1.034 | 0.0375 | 1.633 |
| Hexadecanoic acid | 1.03 | 0.0306 | 1.685 |
| Succinate semialdehyde | 0.942 | 0.0243 | 1.739 |
| 3-Phosphoglycerate | 0.916 | 0.00205 | 2.167 |
| Heptanoic acid | 0.901 | 0.0272 | 1.713 |
| 2,6-ditert-butylphenol | 0.9 | 0.00138 | 2.216 |
| L-Phenylalanine | 0.838 | 0.0173 | 1.814 |
| 11-Eicosenoic acid | 0.782 | 0.0308 | 1.683 |
| Ribitol | 0.667 | 0.0446 | 1.587 |
