# Supplementary material for: Metabolomic Investigation of Ultraviolet Ray-Inactivated White Spot Syndrome Virus-Induced Trained Immunity in Marsupenaeus japonicus
Source: Front Immunol. 2022 May 26;13:885782. doi: 10.3389/fimmu.2022.885782 (PMC9178177; doi:10.3389/fimmu.2022.885782)
Supplement: Supplementary file 6 [file Table_3.docx]

Supplementary Table 3

Primers used in qRT-PCR

| Name | Sequence (5′–3′) |
| --- | --- |
| RT-Vago-L-F | TCACGTGACGGAACCACAAT |
| RT-Vago-L-R | CAGGGCAGCAATCAGGGTAA |
| RT-Ficolin-F | CTACGAGGGCGATGCGAAAT |
| RT-Ficolin-R | CCAACCACCACGATAGACGG |
| β-actin-RT-F | AGTAGCCGCCCTGGTTGTAGAC |
| β-actin-RT-R | TTCTCCATGTCGTCCCAGT |
| VP28-F | AGCTCCAACACCTCCTCCTTCA |
| VP28-R | TTACTCGGTCTCAGTGCCAGA |
